# Automated electrohysterographic detection of uterine contractions for monitoring of pregnancy: feasibility and prospects

**DOI:** 10.1186/s12884-018-1778-1

**Published:** 2018-05-08

**Authors:** C. Muszynski, T. Happillon, K. Azudin, J.-B. Tylcz, D. Istrate, C. Marque

**Affiliations:** 1 Sorbonne Universités, Université de Technologie de Compiègne, CNRS, BMBI UMR 7338, 60200 Compiègne, France; 2 Département de gynécologie et obstétrique, CHU Amiens-Picardie, avenue Laënnec, 80480 Salouël, France

**Keywords:** Electrohysterogram, Automated contraction detection, Uterine contraction, Premature delivery

## Abstract

**Background:**

Preterm birth is a major public health problem in developed countries. In this context, we have conducted research into outpatient monitoring of uterine electrical activity in women at risk of preterm delivery. The objective of this preliminary study was to perform automated detection of uterine contractions (without human intervention or tocographic signal, TOCO) by processing the EHG recorded on the abdomen of pregnant women. The feasibility and accuracy of uterine contraction detection based on EHG processing were tested and compared to expert decision using external tocodynamometry (TOCO) .

**Methods:**

The study protocol was approved by local Ethics Committees under numbers ID-RCB 2016-A00663-48 for France and VSN 02-0006-V2 for Iceland.

Two populations of women were included (threatened preterm birth and labour) in order to test our system of recognition of the various types of uterine contractions.

EHG signal acquisition was performed according to a standardized protocol to ensure optimal reproducibility of EHG recordings. A system of 18 Ag/AgCl surface electrodes was used by placing 16 recording electrodes between the woman’s pubis and umbilicus according to a 4 × 4 matrix. TOCO was recorded simultaneously with EHG recording.

EHG signals were analysed in real-time by calculation of the nonlinear correlation coefficient H^2^. A curve representing the number of correlated pairs of signals according to the value of H^2^ calculated between bipolar signals was then plotted. High values of H^2^ indicated the presence of an event that may correspond to a contraction.

Two tests were performed after detection of an event (fusion and elimination of certain events) in order to increase the contraction detection rate.

**Results:**

The EHG database contained 51 recordings from pregnant women, with a total of 501 contractions previously labelled by analysis of the corresponding tocographic recording. The percentage recognitions obtained by application of the method based on coefficient H^2^ was 100% with 782% of false alarms. Addition of fusion and elimination tests to the previously obtained detections allowed the false alarm rate to be divided by 8.5, while maintaining an excellent detection rate (96%).

**Conclusion:**

These preliminary results appear to be encouraging for monitoring of uterine contractions by algorithm-based automated detection to process the electrohysterographic signal (EHG). This compact recording system, based on the use of surface electrodes attached to the skin, appears to be particularly suitable for outpatient monitoring of uterine contractions, possibly at home, allowing telemonitoring of pregnancies. One of the advantages of EHG processing is that useful information concerning contraction efficiency can be extracted from this signal, which is not possible with the TOCO signal.

## Background

The preterm delivery rate in Europe is between 5.5 and 11.1% and preterm delivery is the leading cause of perinatal morbidity and mortality [1]. Almost one in every two preterm deliveries is the result of spontaneous preterm labour [2]. Preterm delivery rates have remained stable for several years despite the routine use of monitoring tools in clinical practice [3].

In this context, with the support of the SAFE pregnancy@home research project (European Eurostars project), we have conducted research on an outpatient uterine electrical activity monitoring device that could be used for women at risk of preterm delivery. Uterine contraction is the direct result of the electrical activity of the myometrium. Recording of the electrical activity of the myometrium by surface electrodes (electrohysterography [EHG]), a noninvasive technique, can be used to identify changes of the electrical signal related to the type of uterine contraction, Braxton-Hicks contractions of normal pregnancy (inefficient) or labour contractions (efficient) [4–6].

The objective of this preliminary study was to perform automated detection of uterine contractions (without human intervention or TOCO signal using) based on EHG recording and analysis in pregnant women an. The feasibility and accuracy of electrohysterographic detection of contractions was compared to those of external tocodynamometry (TOCO).

## Methods

### Patient characteristics

Pregnant women, included in two university hospitals (France and Iceland, Icelandic database [7]) between 2015 and 2017, were classified into two categories. The first group, the Pregnancy group, consisted of women at 26 to 35 weeks of gestation (WG), during their normal pregnancy follow-up, or admitted for threatened preterm labour. Admissions for threatened preterm labour were defined by the presence of contractions experienced by the woman and/or present on tocography, associated with cervical changes, i.e. short cervical length on pelvic examination or vaginal ultrasound measurement (transvaginal cervical length < 25 mm). The second group, the Labour group, consisted of women in labour at term, defined by at least 4 cm of cervical dilatation associated with regular uterine contractions. Women in were recorded either at the first stage or second stage of labour but none of them were recorded during the pushing stage. These two populations of women were included in order to test our system of recognition of the various types of uterine contractions (Braxton-Hicks contractions and labour contractions). Women with pathological pregnancies (maternal or obstetric disease) other than threatened preterm labour were not included in this study. All women were over the age of 18 years, with a BMI < 35, covered by national health insurance in the country concerned, and provided their written informed consent to participate in the study and to publish data and results in scientific journals. The study protocol was approved by local Ethics Committees under numbers ID-RCB 2016-A00663-48 for France and VSN 02-0006-V2 for Iceland.

### Electrohysterography

In each centre, informed consent was obtained from each woman by the same person who also performed EHG recordings.

**Fig. 1:**
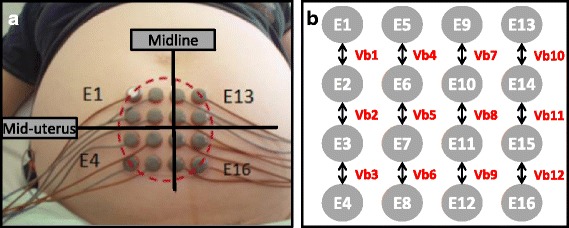
**a** Positioning of the 16 electrodes (Ei, i ϵ [1–16]) according to a 4 × 4 matrix. This matrix is shifted slightly towards the right due to the physiological dextrorotation of the uterus. **b** Twelve bipolar signals are obtained (Vbi, i ϵ [1–12]) by vertical subtraction of 2 adjacent unipolar signals

EHG signal acquisition was performed according to a standardized protocol to ensure optimal reproducibility of EHG recordings in the two maternity units [7]. Before applying the electrodes, the woman’s skin was prepared by applying exfoliating cream to reduce inter-electrode impedance and to increase the signal-to-noise ratio of the recorded signals. A system of 18 Ag/AgCl surface electrodes was used by placing 16 recording electrodes between the woman’s pubis and umbilicus according to a 4 × 4 matrix and two reference electrodes on each of the woman’s hips. The 4 × 4 electrode matrix was shifted slightly to the right due to physiological dextrorotation of the uterus, as indicated in Fig. 1a. The sixteen 13-mm diameter electrodes were separated from each other by intervals of 17.5 mm (centre to centre). Electrical signals were recorded at a sampling frequency of 200 Hz and were then transferred by Wifi to a laptop computer for processing by TMSi PolyBench® software. All components of the EHG signal recording system are CE marked.

### Signal preprocessing

The 16 unipolar signals distributed over the 4 × 4 matrix were subtracted two by two, according to the vertical axis, to obtain 12 bipolar signals, as indicated in Fig. 1b (Vbi, *i* = 1 to 12). Analysis of bipolar electrical signals has the advantage of increasing the signal-to-noise ratio and consequently increasing the signal quality. A bandpass filter was then applied to only select the 0.1-3 Hz frequency band, corresponding to the frequency spectrum of uterine contractions recorded by electrohysterography [8].

### Characterization of series of uterine contractions

In this study, all EHG signals of uterine contractions were collected simultaneously with external tocodynamometry (TOCO) recordings. Although the accuracy of external TOCO has been reported to be poor, external TOCO was the only reference examination available in this study for experts to identify the presence of a contraction and to define the start and end times of EHG signals, while reviewing all signals. The start and end times of each contractile event, obtained by this peer review of TOCO and EHG signals, were used as the basis to test the feasibility and performance of algorithm-based automated EHG detection of uterine contractions.

### Application of the nonlinear correlation coefficient H^2^

**Fig. 2:**
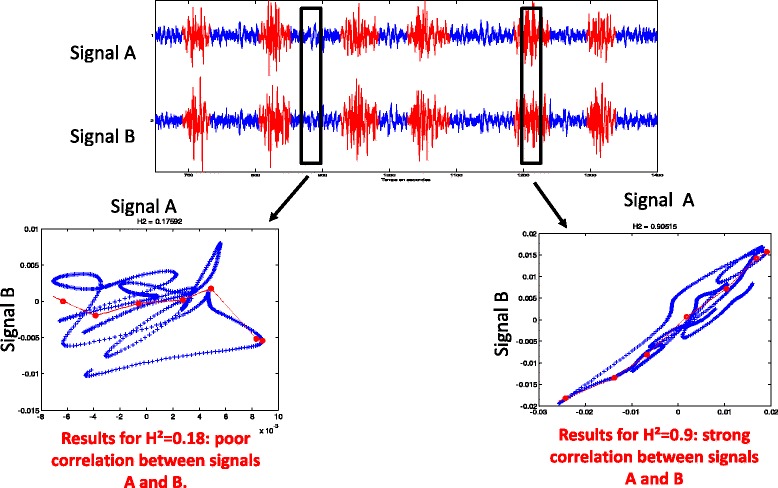
Real-time EHG analysis by application of the nonlinear correlation coefficient H^2^ to two adjacent bipolar signals. The analysis on the left concerns an EHG segment with no contractions (bottom line) associated with a poor correlation between the signals (H^2^ = 0.18). The analysis on the right concerns an EHG segment during a contraction associated with a strong correlation between the signals (H^2^ = 0.9)

EHG signals were analysed in real-time by calculation of the nonlinear correlation coefficient H^2^. Each H^2^ value was situated between 0 (non-correlated signals) and 1 (strongly correlated signals), as, in a previous study [9], we observed that EHG signals were correlated with high H^2^ values during uterine contractions, while low H^2^ values were observed for baseline signals between two contractions (Fig. 2). A sliding window, with a width equal to 800 acquisition points (i.e. 4 s), scanned the 12 bipolar EHG signals with a 400-point increment. At each position of this window, 36 H^2^ values were calculated from the 36 laterally adjacent pairs of bipolar signals (for example between Vb1 and Vb4, between Vb4 and Vb7, between Vb2 and Vb5, etc.). A first cutoff for H^2^, S1, was tested and defined to determine whether or not a pair of bipolar signals can be considered to be correlated. For each position of the sliding window, the number of H^2^ values greater than S1 was then counted (corresponding to the number of correlated pairs among the 36 pairs tested for each window position), resulting in a curve representing the number of correlated pairs of signals for each position of the sliding window.

### Detection of contractions with H^2^

**Fig. 3:**
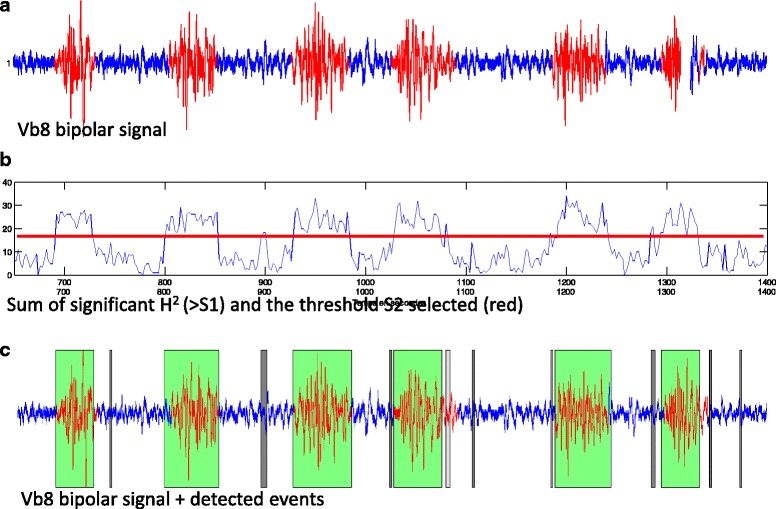
**a** In red: electrical activity associated with a contraction previously identified on concomitant tocography (reference contraction). **b** Curve of calculated H^2^ values and the cutoff adopted beyond which an event is validated. **c** Detected events: all contractions were fully or partly detected (green), but some detected events do not correspond to contractions and were considered to be false alarms (gray)

The values of the curve obtained in the previous step were compared according to a second cutoff, S2, corresponding to the limit of detection. H^2^ values higher than S2 indicate the presence of an event (i.e. a sufficient number among the 36 pairs of signals are correlated over a given interval) (Fig. 3).

### Fusion and elimination

Two tests were performed after the detection of events. The first test studied the interval between one event and the previous event. These two events were then fused when they were situated sufficiently close to each other (i.e. separated by a difference less than a tested and defined value, D1).

The second test studied the duration of the events detected. An event was eliminated when it did not exceed a minimal duration tested and defined in this study (D2).

### Methodology of evaluation of the performance of automated detection of uterine contractions

**Fig. 4:**
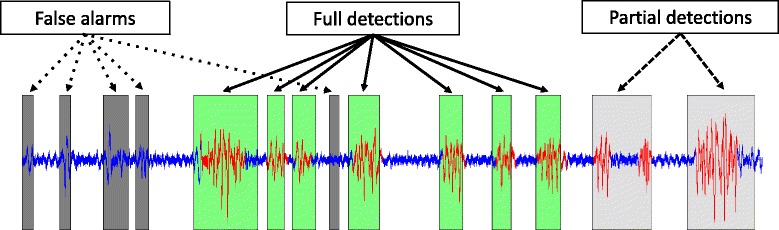
Fully detected contraction (green): contraction, for which the segment detected by the algorithm corresponds (which a short time difference) to the limits indicated by the experts; Partial detection (light grey): detection of only a part of the contraction, or comprising noise in the detected segment; False alarm (dark grey): an event that is detected but does not correspond to any reference contraction

All events detected for each recording were then compared to the reference labels of contractions previously identified by the experts. Three situations were observed: i) an event was detected in the absence of any identified contraction, and was therefore considered to be a “False alarm”; ii) an event was detected during a contraction and its start and end limits coincided with the reference labels (within an acceptable margin), and was therefore considered to be a “Full detection”; iii) when the limits of the event did not coincide with the reference labels, but covered only a part of the duration of the contraction or contained the baseline signal, it was therefore considered to be a “Partial detection” (Fig. 4).

## Results

The EHG database contained 51 recordings from pregnant women, with a total of 501 contractions previously labelled by analysis of the corresponding tocographic recording. The percentages and number of recognitions obtained (full detections, partial detections and false alarms) by application of the first step of the method based on coefficient H^2^ only are presented in Table 1.

**Table 1:** Results of automated detection of uterine contractions by analysis based on the nonlinear correlation coefficient H^2^ alone. The database used contains 501 labelled contractions

Full detections	Partial detections	Total detections	False alarms
62.5% (313/501)	37.5% (188/501)	100% (501/501)	782% (3918/ 501)

The results obtained after adding fusion and elimination steps to the previously obtained detections are shown in Table 2.

**Table 2:** Results of automated detection of uterine contractions after the addition of fusion and elimination steps. The database used contains 501 labelled contractions

Full detections	Partial detections	Total detections	False alarms
63% (316/501)	30.7% (154/501)	92.6% (464/501)	92.6% (464/ 501)

The automated detection algorithm based on H^2^ coefficients, followed by fusion and elimination steps, detected 96% of contractions (485 contractions out of a total of 501 contractions), with 62% of full detections. As explained previously (Fig. 4), a fully detected contraction is a contraction for which the segment detected by the algorithm corresponds (with a short time difference) to the limits indicated by the experts; a “partial detection” was noted when only part of the contraction or some noise was included in the detected segment. The number of false alarms (segments detected that did not correspond to a contraction identified by the experts) was 496 for 501 real contractions present on the EHG signals.

## Discussion

Algorithm-based (automated) detection of uterine contractions is a major prerequisite to the use of EHG for the monitoring of at-risk pregnancies. The use of EHG has several advantages over tocography. Application of EHG electrodes adherent to the woman’s skin avoids the problems of electrode displacement, in contrast with the tocodynamometer, which must always be correctly maintained by a belt in order to detect uterine contractions. External tocography, can be used to evaluate intrauterine pressure by recording deformation of the uterine fundus across the maternal abdominal wall [10]. Displacement or poor positioning of the external pressure transducer can therefore modify the signals recorded during contraction. Internal tocography has therefore been developed to overcome the lack of precision of external tocography, mainly in terms of quantification of the amplitude of contraction [11]. However, internal tocography requires rupture of the amniotic membranes and is therefore an invasive procedure that can only be performed during labour. Electrohysterographic recording of uterine activity was therefore developed in this context. Electrohysterography is an appropriate tool to evaluate intrauterine pressure and consequently the mechanical effect of a contraction using surface electrodes [12]. It is therefore a noninvasive procedure that can be used to monitor contractions during pregnancy. The present study used a small signal recording system that can be attached to the woman’s hip, facilitating outpatient monitoring, in contrast with external tocography, which usually requires immobilization of the patient next to the apparatus. EHG allows mobilization during recording, making it a particularly attractive tool for outpatient monitoring, possibly at home. Another advantage of EHG is that, in the presence of contractions, it is able to discriminate between labour contractions and Braxton-Hicks contractions, and EHG analysis during pregnancy can be used to predict the risk of labour [13–18]. EHG could therefore be a potentially useful tool for the monitoring of patients with threatened preterm labour.

The automated detection of contraction based on EHG processing was compared to reference labels of contractions identified by experts using TOCO in order to evaluate the performance of detection of uterine contractions by EHG signals. Although the use of TOCO has not been able to decrease the premature delivery rate over recent years, it can be used to diagnose the number of contractions. However, TOCO does not provide any information on the type of contraction (physiological or pathological) in contrast with EHG analysis. Although intrauterine pressure determination appears to be slightly more accurate than EHG to detect uterine contraction, this device cannot be widely used during labour (risk of infections) and cannot be used during pregnancy with intact membranes [19]. To evaluate the accuracy of contraction detection in clinical practice, we compare EHG signal detection with the only device currently available in routine clinical practice, TOCO.

However, continuous automated detection of EHG spikes associated with uterine contractions must first be performed before this tool can be widely used in clinical practice in hospital or at home. The first studies concerning EHG detection of contractions, performed in the early 2000s, used a method based on the frequency content of the EHG signal [20]. The preliminary results of our study on algorithm-based automated detection of uterine contractions by EHG based exclusively on correlation coefficient H^2^ showed a high level of accuracy of detection, but with a very high false alarm rate (3918 false alarms for 501 contractions), which can be explained by the fact that this method of detection is based on interpretation of the various correlations observed for the signals throughout signal acquisition. However, a strong correlation observed between signals recorded at a given point in time is not necessarily related to a uterine contraction, as these signals may also be due to active foetal or maternal movements or an instrumental artifact (movement of electrode leads, etc.). Consequently, a large proportion of the events detected by the correlation coefficient H^2^ alone are due to these foetal, maternal or instrumental artifacts. In the present study, these events were considered to be false alarms. Furthermore, the information provided by the EHG signal during a contraction is not homogeneous, as some contractions may be only partially detected or may be divided into several events (oversegmentation), resulting in detection of several events for a single uterine contraction. Inversely, the contraction detection rate (complete and partial detection) was 100%, which confirms that a strongly correlated electrical activity was responsible for the mechanical activity of the uterus. In this study, in order to decrease the number of false alarms, we have developed a technique of fusion and elimination of events according to their duration and the time intervals between events. Events closely related in time were fused, as they are considered to correspond to the same contraction, thereby decreasing oversegmentation (reduction of the number of events during the same contraction). Event fusion therefore increases the number of complete detections by decreasing the number of partial detections. Elimination of brief events decreased the number of events related to brief foetal or maternal movements, but also eliminated brief events detected within a contraction that were not fused during the previous step. This procedure resulted in a reduction of the false alarm rate (from 3918 to 496), as well as the short partial detection rate. Overall, this fusion-elimination technique allowed a ninefold reduction of the number of false alarms, while maintaining a very high uterine contraction detection rate of 96% (485 contractions detected out of 501).

However, the current false alarm rate obviously needs to be further decreased to allow effective clinical application of automated detection of uterine contractions by EHG. A study is currently underway in order to link this method of analysis based on correlation of EHG signals with a method based on EHG wavelet decomposition, taking frequency data into account. Linking of these two methods is designed to further decrease the false alarm rate, while also making detection of contractions more reliable, based on both their correlation and their frequency content. EHGs recorded during contractions, rest phases or in the presence of artifacts, present different characteristics in terms of correlation (demonstrated by correlation coefficient H^2^), as well as frequency content (demonstrated by wavelets). The combination of these two methods should improve the accuracy of detection of uterine contractions. A final step would need to consider other parameters (temporal and/or frequency) already shown to be potentially relevant to the analysis of EHG signals [19], and which could allow optimal reduction of the false alarm rate. Finally, in order to make a decision concerning the EHG bursts identified by the algorithm and not associated with a TOCO event (currently considered to be false alarms), we are presently post-processing the false alarms EHG bursts to see whether their characteristics are similar to those of the EHG bursts associated with a TOCO event. If this is the case, these events will subsequently be considered to be contractions, and will be used for clinical diagnosis. If their characteristics differ, they will remain classified as false alarms.

After ensuring reliable detection of uterine contractions, this EHG database, recorded at various terms of pregnancy and during labour, could then be used to determine whether EHG is able to discriminate between these various events. We would therefore be able to study EHG parameters specific to the type of contractions during pregnancy (physiological or pathological) and during labour, allowing real-time estimation of the intensity of each contraction detected and therefore the risk of preterm delivery. Apart from simple counting of uterine contractions, EHG monitoring of pregnant women would allow estimation of the efficiency of these contractions, thereby providing an estimation of the risk of subsequent labour over the days following the recording. This objective constitutes the subject of ongoing studies on available and further EHG databases.

## Conclusions

These preliminary results appear to be encouraging for the diagnosis and algorithm-based automated monitoring of uterine contractions by electrohysterography (EHG). We are currently pursuing our research in order to reduce the number of false alarms, while maintaining, or even increasing, the good detection rates obtained in this study.

Finally, this compact recording system, comprising surface electrodes attached to the skin, appears to be particularly suitable for outpatient monitoring of uterine contractions, possibly at home, allowing telemonitoring of pregnancies.

## Abbreviations

EHG: Electrohysterography
H^2^: Nonlinear correlation coefficient
TOCO: External tocodynamometry
WG: Weeks of gestation
